# The relationship between psychological distress, depressive symptoms, emotional eating behaviors and the health-related quality of life of middle-aged korean females: a serial mediation model

**DOI:** 10.1186/s12912-023-01303-y

**Published:** 2023-04-23

**Authors:** Jihyun Oh, Sunghee Kim

**Affiliations:** 1 Department of Nursing, College of Nursing and Health, Kongju National University, Kongju, 32588 South Korea; 2 Red Cross College of Nursing, Chung-Ang University, 84 Heukseok-Ro, Dongjak-Gu, Seoul, 06974 South Korea

**Keywords:** Psychological distress, Quality of life, Mental health, Depressive symptoms, Women

## Abstract

**Background:**

This study aimed to examine the effects of psychological distress, depressive symptoms, and emotional eating behaviors on the health-related quality of life of middle-aged Korean females. This study provides primary data for developing an intervention program to improve the health-related quality of life of middle-aged females.

**Methods:**

Middle-aged females between 35 and 64 years old, from July 22 to August 10, 2021, were included in this study. The mediating effects of depressive symptoms and emotional eating behaviors on the relationship between psychological distress and health-related quality of life were investigated. A cross-sectional survey was conducted on 325 subjects. Researchers conducted questionnaires measuring psychological distress, depressive symptoms, emotional eating behavior, and health-related quality of life.

**Results:**

The findings of this study demonstrated a correlation between the severity of a female’s depressive symptoms and the degree of their psychological distress, indicating that severe depressive symptoms were associated with negative emotions, which increased emotional eating behaviors. Additionally, more severe depressive symptoms indicated a lower health-related quality of life. Higher psychological distress was associated with increased emotional eating behaviors and lower health-related quality of life. The total and direct effects of psychological distress on the health-related quality of life were statistically significant.

**Conclusions:**

In this study, psychological distress, depressive symptoms, and emotional eating behaviors affected the health-related quality of life of middle-aged Korean females. This study also confirmed that psychological distress had a direct effect on health-related quality of life. These findings serve as primary data for evidence-based intervention programs that alleviate emotional health problems, such as psychological distress and depressive symptoms in middle-aged females. Moreover, nurses can help develop effective treatment strategies to improve health-related quality of life by identifying and assessing potential symptoms of psychological distress, depressive symptoms, and emotional eating behaviors.

## Introduction

Middle age is a stage of decline among the developmental stages of life. Also, this stage is a period of many physical, mental, and social changes and challenges and changes in various life roles [1]. For Females, middle age is a period of menopause and aging due to changes in the life cycle [2]. In particular, menopause results in a sense of loss as females age and experiences a decline in physical functions due to hormone changes [3]. Middle-aged Korean females range from 35 to 64 years old [4]. In addition, the weakening of ties within the family and the reduction of social networks due to the growth and independence of their children lead to feelings of alienation and social and psychological developmental crises [2]. If middle-aged females in this period adequately cope with the various physical changes, it will positively affect their quality of life after middle adulthood [1]. However, if middle-aged females experience physical, psychological, or emotional confusion, it will negatively affect their health-related quality of life [3].

Looking at the demographic age trend of Korean females, the proportion of females aged 40 to 60 out of the total female population was the highest at 32.3% in 2020. This percentage has continuously increased since 2010 when it was 30.8% [5]. There is a high incidence of depressive symptoms among middle-aged females in Korea due to the psychological distress of the increased responsibilities and conflicts between them and their adult children, changes in physical aging, and maladaptation to the loss of their physiological ability [6]. The psychological distress experienced by middle-aged females can cause physical and mental symptoms, such as peripheral vascular disorder, gastrointestinal discomfort, muscle tension, and depression [7]. This psychological distress can harm their physical and mental health resulting in a lower health-related quality of life [8, 9]. Therefore, it is important to examine how psychological distress affects the health-related quality of life of middle-aged females in Korea.

Health-related quality of life is a subjective evaluation of health-related factors, including overall health, physical functions, body role, pain, vitality, and social and mental functions [10], and is defined as the meaning of life in terms of health and functional status [11]. Health-related quality of life is a comprehensive health state with a high correlation to diseases and physical conditions [12].

The health-related quality of life of middle-aged females is an indicator that reflects their daily functioning and well-being. It has been reported that the health-related quality of life of middle-aged females is affected by psychological stress, social support, and dietary habits [9, 13]. The higher the psychological distress experienced by middle-aged females, the higher the emotional states of depression-disappointment, tension-anxiety, and anger-hostility [7]. Additionally, the most common symptoms experienced by middle-aged females are depressive symptoms, anxiety, and nervousness [14]. Depressive symptoms are a typical mental health problem for middle-aged Korean females. It has been reported that 13.7% of Korean females have had depressive symptoms, about twice as high as the percentage of Korean males (6.6%) [15]. A previous study reported that depressive symptoms in middle-aged females lowered their health-related quality of life [14]. These results indicate that plans to improve the health-related quality of life through the continuous management of depressive symptoms are required.

Meanwhile, psychological distress and stress have been associated with dietary behaviors affecting dietary intake patterns, inducing overeating and meal-skipping [16]. In addition, excessive psychological distress can lead to eating disorder behaviors, which can lead to psychological pressure and eating disorders, such as anorexia and bulimia [17]. Emotional eating is an eating disorder characterized by the tendency to overeat in response to stress or negative emotions [18, 19]. Emotional eating behaviors induced by emotional stimuli can cause changes in individuals’ dietary habits and can result in health problems [20, 21]. Emotional eating behaviors can cause a loss of balance in daily eating habits and negatively affect mental and physical health, leading to a decrease in health-related quality of life.

It has been reported that the health-related quality of life of middle-aged Korean females was lower than that of male adults and females of other age groups [13]. Therefore, it is necessary to pay attention to the health-related quality of life of middle-aged Korean females.

It is necessary to understand that psychosocial factors, such as stress, social support, and lifestyle, can affect the health or health-related quality of life of middle-aged Korean females [22]. However, few studies have comprehensively investigated the relationship between psychological distress and the health-related quality of life of middle-aged Korean females using depressive symptoms and emotional eating behaviors as a medium.

Therefore, this study aimed to examine the effects of psychological distress, depressive symptoms, and emotional eating behaviors on the health-related quality of life of middle-aged Korean females.

### Materials & methods

#### Design

This study was a cross-sectional survey conducted on middle-aged females aged between 35 and 64 years old from July 22 to August 10, 2021. This study investigated the mediating effects of depressive symptoms and emotional eating behaviors on the relationship between psychological distress and health-related quality of life.

### Participants

The researchers who conducted this study visited three community centers in Seoul to obtain in-person participation consent from middle-aged females who could communicate and understand the purpose of this study. Participants had to be middle-aged between the ages of 35 and 64 years old and female who could read and understand the details of the informed consent forms and reside in Seoul. The researchers retrieved a total of 268 questionnaires from the three community centers, of which 257 were included in this analysis (response rate: 96.7%). There were 11 questionnaires that had missing data, and these questionnaires were excluded. Calculations were made using the G*Power 3.1 analysis software with a significance level of 0.05, a median size of regression analysis of 0.15, and a power of 0.95 to confirm the suitability of the sample size. The minimum number of subjects was calculated to be 129 [23]. Given that this study included 257 subjects, the number of subjects was found to be adequate. A larger sample size in a study can increase the reliability and the possibility of generalizing the research results. Therefore, exceeding the minimum required number of participants is a positive aspect of this study.

### Measurement

#### Psychological distress

In this study, the 12-Item General Health Questionnaire (GHQ-12), a self-reported scale developed by Goldberg and Williams [24]and revised by Park et al. [25], was used to screen and measure the early discovery of minor psychiatric disorders and psychological distress. The questionnaire asked the respondents about their experiences with psychological conditions in the preceding two to three weeks to identify current issues. Responses were measured on a 4-point Likert scoring scale (from 0 = not at all to 3 = more than usual), with a total score of 0 to 36. A higher total score indicated greater psychological distress. The Cronbach’s α of the Korean version of the GHQ-12 [25]was 0.88. This version shows a Cronbach α of 0.92, indicating higher reliability.

#### Depressive symptoms

The Patient Health Questionnaire-9 (PHQ-9), developed by Kroenke et al. [26], is a reliable and validated tool that can be used for screening, diagnosing, monitoring, and evaluating the severity of depression symptoms. However, The PHQ-9 scale only measures “depressive symptoms” and not “depression.“ The PHQ-9 consists of nine items corresponding to the diagnostic criteria of major depressive disorders according to the Diagnostic and Statistical Manual of Mental Disorders, Fourth Edition (DSM-IV; 2013 Edition). The PHQ-9 is a self-reported test created for screening for depressive symptoms in the preceding two weeks and assessing the severity of the depressive symptoms. Each question on the PHQ-9 was scored on a 4-point scale ranging from 0 (not at all) to 3 (nearly every day). The total scores ranged from 0 to 27 and were obtained by adding the scores of all nine items. Higher scores indicated higher depressive symptoms. PHQ-9 scores of 5, 10, 15, and 20 were categorized as the cut-off values for no depressive symptoms (0–4), mild depressive symptoms (5–9), moderate depressive symptoms (10–14), moderately severe depressive symptoms (15–19), and severe depressive symptoms (20–27) [27]. Overall, the PHQ-9 is a valuable tool for assessing depressive symptoms and can help clinicians and researchers identify and monitor depressive symptoms. Kroenke et al. [26]have shown that the PHQ-9 scale has good validity and reliability. In this study, Cronbach’s α was 0.93, indicating excellent reliability.

#### Emotional eating behavior

Emotional eating behaviors were measured using the Dutch Eating Behavior Questionnaire (DEBQ), consisting of 13 questions, and scored on a 5-point scale ranging from 1 (never) to 5 (very often) [28]. The DEBQ was originally developed as 33 items within three factors. This study only measured one factor, emotional eating, out of the three factors (restrained eating, emotional eating, and external eating). Emotional eating is eating in response to negative emotions. The Emotional Eating subscale consists of 13 questions, including “Do you have the desire to eat when you are irritated?” Higher scores indicate a higher tendency toward emotional eating behaviors. van Strien et al. [28]reported that the DEBQ has good reliability and validity. The Cronbach’s α was 0.97 for the emotional eating subscale [29], and the Cronbach’s α in this study was 0.90, indicating high reliability.

#### Health-related quality of life

The 12-Item Short Form Health Survey (SF-12) was developed by Ware et al. [30]. The physical components summary scale (PCS) consists of four subdomains, physical functioning (PF), role-physical (RP), body pain (BP), and general health (GH). The mental components summary scale (MCS) consists of four subdomains, mental health (MH), role emotion (RE), social functioning (SF), and vitality (VT). A higher PCS score indicates a better physical health-related quality of life, while a higher MCS score indicates a better mental health-related quality of life. The raw scores were converted to scores between 0 (the worst) and 100 (the best). The SF-12 has been shown to be reliable and valid for use in middle-aged and older adults [31]. The Cronbach’s α in this study was 0.87, indicating good reliability.

### Data collection

This study was conducted at three community centers from July 22 to August 10, 2021. Data were collected from middle-aged females using a structured questionnaire. The researchers distributed the questionnaires to the participants in person, and the participants returned them to the researchers upon completion. The researchers provided sufficient information to the potential participants about the study, including its purpose, methods, and any potential risks or benefits. After the participants had fully understood this information, they were asked to provide their consent in writing, indicating that they voluntarily agreed to participate in the study. The questionnaire took approximately 20 min to complete.

### Ethical considerations

This study was approved by the Institutional Review Board at the University (No. 1040647-202105-HR-002-02). All participants were provided with detailed information on the purpose and process of this study and were provided with a written consent form before completing the questionnaire. The questionnaire was provided only to the participants in this study. The participants voluntarily participated, and data obtained from the questionnaires were not used for any purposes other than those of this study. Participants could withdraw from this study at any time without incurring any repercussions.

### Statistical analyses

The analyses of the data of this study were conducted using the SPSS 27.0 statistical software (version 23; IBM Corp, Armonk, New York, USA) and SPSS PROCESS Macro (version 3.5) [32]. Frequency and descriptive statistical analyses were performed to identify the general characteristics and characteristics of the major variable. Independent *t*-tests and ANOVAs were conducted to check for differences in the health-related quality of life according to the general characteristics. A Scheffé’s test was conducted for the post-hoc analysis. A Pearson correlation analysis was performed to confirm the correlation between each variable. The serial multiple mediating effects of depressive symptoms (M1) and emotional eating behaviors (M2) on the relationship between psychological distress (X) and health-related quality of life (Y) were verified. The serial mediation analysis was performed using SPSS Macro Process Model 6 developed by Hayes [32], which we identified as the most appropriate verification method for this study. All effects were reported with 95% confidence intervals (CIs). The mediating effect was analyzed with a 95% CI to test the significance using the bootstrapping method with 5,000 resampled data; if 0, the mediating effect was not included.

## Results

### General characteristics and health-related quality of life according to the general characteristics

Table 1 shows the general characteristics of the participants. Among the 268 questionnaires retrieved, 11 had incomplete responses and were excluded. A total of 257 subjects were included in the analysis (response rate: 98.0%). The majority (56.4%) of the participants were between the ages of 40 and 49 years old; the median age was 45.76 years. Most of the participants were married (77.8%), non-drinkers (58.8%), non-smokers (94.2%), did not engage in regular exercise (53.7%), had a college-level education (77.8%), had an economic status in the median range (79.4%), and did not have a chronic disease (60.7%). The most common type of chronic illness was musculoskeletal disorders, such as disc and arthritis, accounting for 23.6%. There was a significant difference in the total health-related quality of life scores according to marital status (*F* = 4.441, *p* = .013).

**Table 1:** General characteristics of the subjects *(n = 257)*

Variable	Category	*n* (%)	Health-related quality of life
Age, mean (*SD*)			45.76 (7.67)
Age groups (years)	35–39	49 (19.1)	65.63 (6.62)
(35–64)	40–49	145 (56.4)	64.12 (7.84)
	50–59	48 (18.7)	65.91 (6.05)
	≥ 60	15 (5.8)	64.00 (8.95)
*F(p)*			1.037 (0.377)
Marital status	Single	43 (16.7)	67.66 (7.11)
	Married	200 (77.8)	64.25 (7.04)
	Divorced or Widowed	14 (5.4)	62.71 (7.38)
*F(p)*			4.441 (0.013)
Alcohol	Yes	106 (41.2)	64.88 (7.22)
	No	151 (58.8)	64.63 (7.52)
*t(p)*			0.263 (0.793)
Smoking	Yes	15 (5.8)	62.50 (10.82)
	No	242 (94.2)	64.87 (7.13)
*t(p)*			-1.206 (0.229)
Regular exercise	Yes	119 (46.3)	64.64 (7.23)
	No	138 (53.7)	64.81 (7.54)
*t(p)*			-0.184 (0.854)
Education	≤ High school	57 (22.2)	64.86 (8.22)
	≥ College	200 (77.8)	64.70 (7.15)
*t(p)*			0.149 (0.882)
Economic status	High	17 (6.6)	61.33 (8.49)
	Middle	204 (79.4)	64.98 (7.07)
	Low	36 (14.0)	64.98 (8.38)
*F(p)*			1.947 (0.145)
No. of chronic disease	0	156 (60.7)	65.19 (7.78)
	1	89 (34.6)	63.66 (6.88)
	≥ 2	12 (4.7)	66.74 (4.54)
*F(p)*			1.685 (0.188)
Types of chronic disease	Hypertension	14 (12.7)	
(Multiple responses)	Cardiovascular disease	7 (6.4)	
	Musculoskeletal disorders	26 (23.6)	
	Depressive symptoms	7 (6.4)	
	Diabetes	4 (3.6)	
	High cholesterol	13 (11.8)	
	Respiratory disorders (asthma, bronchitis)	2 (1.8)	
	Others	37 (33.6)	

*SD* = standard deviation;

### Levels of depressive symptoms, psychological distress, emotional eating behaviors, and health-related quality of life

Table 2 shows the mean score of each variable. The mean depressive symptoms score was 6.28 points, indicating mild depressive symptoms. The mean psychological distress score was 13.55, and the mean emotional eating behaviors score was 27.63. The mean total health-related quality of life score was 64.74 out of 100 points, the mean MCS was 69.58, and the mean PCS was 59.89.

**Table 2:** Levels of the variables *(n = 257)*

Variables	Range	Mean (*SD*)
Depressive symptoms	0–25	6.28 (4.87)
Psychological distress	4–29	13.55 (4.57)
Emotional eating behavior	13–65	27.63 (11.33)
Total health-related quality of life	37.07–81.38	64.74 (7.38)
MCS	43.33–96.67	69.58 (9.19)
PCS	24.14–82.76	59.89 (7.64)

*SD* = standard deviation; MCS = mental components summary scale; PCS = physical components summary scale.

### Correlation between variables

Table 3 shows the correlations among depressive symptoms, psychological distress, emotional eating behaviors, and health-related quality of life in the participants of this study. Depressive symptoms were positively correlated with psychological distress (r = .657, *p* < .001) and emotional eating behaviors (r = .321, *p* < .001). This correlation indicated that the more severe the depressive symptoms, the worse a female’s psychological distress and that severe depressive symptoms were associated with negative emotions, which increased emotional eating behaviors. Depressive symptoms were also significantly negatively correlated with health-related quality of life (r = − .283, *p* < .001). A more severe depressive symptoms score indicated a lower health-related quality of life. A statistically significant positive correlation was shown between psychological distress and emotional eating behaviors (r = .290, *p* < .001). A statistically significant negative correlation was shown between psychological distress and health-related quality of life (r = − .348, *p* < .001). This correlation indicated that higher psychological distress was associated with increased emotional eating behaviors and a lower health-related quality of life. A statistically significant negative correlation was shown between emotional eating behaviors and health-related quality of life (r = − .222, *p* < .001). In conclusion, the health-related quality of life decreased as emotional eating behaviors increased.

**Table 3:** Correlations among the variables *(n = 257)*

Variables	Depressive symptoms (*p-*value)	Psychological distress (*p-*value)	Emotional eating behavior (*p-*value)	Health-related quality of life (*p-*value)
Depressive symptoms	―			
Psychological distress	0.657 (< 0.001)	―		
Emotional eating behaviors	0.321 (< 0.001)	0.290 (< 0.001)	―	
Health-related quality of life	− 0.283 (< 0.001)	− 0.348 (< 0.001)	− 0.222 (< 0.001)	―

### Mediating effects of depressive symptoms and emotional eating behaviors in the relationship between psychological distress and health-related quality of life

**Fig. 1:**
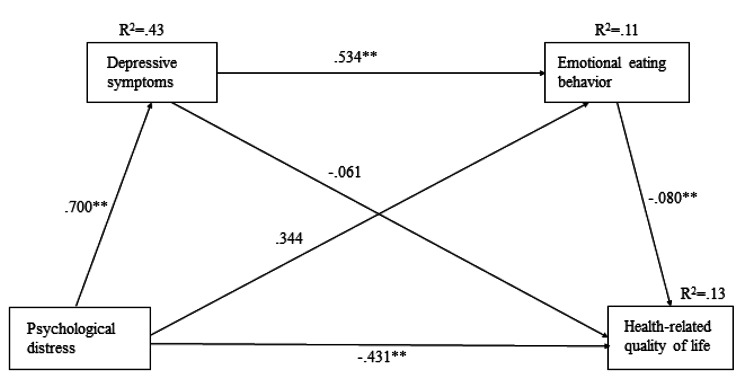
The results of the multiple mediation model testing for depressive symptoms and emotional eating behaviors as mediators of the effect of psychological distress on health-related quality of life. ** *p* < .01

The SPSS PROCESS Macro Model 6 suggested by Hayes [32] was used to verify the mediating effects of depressive symptoms and emotional eating behaviors in the relationship between psychological distress and health-related quality of life among the participants of this study, and the results of the standardized path coefficients are presented in Fig. 1. The results of the serial multiple mediation analysis are shown in Table 4. The effects of psychological distress on the health-related quality of life were statistically significant (*B* = − 0.3318, *SE* = 0.0561, *t* = -5.9201, *p* < .001). In addition, the direct effects of psychological distress on the health-related quality of life were statistically significant (*B* = − 0.2552, *SE* = 0.0743, *t* = -3.4344, *p* = .0007). The increased psychological pain directly translated to decreased health-related quality of life. However, the indirect effect of depressive symptoms on the mediating variable (M1) (*B* = − 0.0427, *SE* = 0.0571, 95% BC CI [-0.1622, 0.0630]) and the indirect effect of emotional eating behaviors on the mediating variable (M2) (*B* = − 0.0177, *SE* = 0.0134, 95% BC CI [-0.0492, 0.0025]) were not statistically significant in the relationship between psychological distress and health-related quality of life. Moreover, the bootstrapped indirect effect of emotional eating behaviors on the mediating variable (M2) (*B* = − 0.0162, *SE* = 0.0135, 95% BC CI [-0.0464, 0.0062]) and the bootstrapped indirect effect of depressive symptoms on the mediating variable (M1) were found not to be statistically significant in the relationship between psychological distress and the health-related quality of life. The total indirect effect on the mediating variables was insignificant. Therefore, reducing psychological distress may directly enhance the health-related quality of life. In sum, depressive symptoms and emotional eating behaviors partially mediated the relationship between psychological distress and health-related quality of life.

**Table 4:** Total, direct, and indirect effects for a multiple mediator model *(n = 257)*

Model	Effect	*SE*	*t-*test	*p-*value	*95% BC CI*
Total effect of psychological distress on HRQOL	− 0.3318	0.0561	-5.9201	< 0.001	[-0.4422, − 0.2214]
Direct effect of psychological distress on HRQOL	− 0.2552	0.0743	-3.4344	0.0007	[-0.4015, − 0.1089]
Total indirect effect	− 0.0766	0.0606			[-0.2011, 0.0363]
Indirect effect via					
Depressive symptoms	− 0.0427	0.0571			[-0.1622, 0.0630]
Depressive symptoms and emotional eating behaviors	− 0.0177	0.0134			[-0.0492, 0.0025]
Emotional eating behaviors	− 0.0162	0.0135			[-0.0464, 0.0062]

SE = standard error; BC CI = bias-corrected confidence interval; HRQOL = health-related quality of life

## Discussion

This study was conducted to investigate the relationship and effects of psychological distress, depressive symptoms, and emotional eating behaviors on the health-related quality of life of middle-aged Korean females. This study found that the health-related quality of life of middle-aged Korean females was 64.74 out of 100, indicating mild depressive symptoms. The more severe the depressive symptoms, the more severe the individual’s psychological distress, and these negative emotions were found to increase emotional eating behaviors. Depressive symptoms showed a negative correlation with health-related quality of life. The higher the psychological distress, the higher the depressive symptoms and emotional eating behaviors, and the lower the health-related quality of life.

According to the general characteristics of this study, the health-related quality of life of middle-aged Korean females was found to be significantly different according to marital status. Personal factors that affected the overall quality of life included health-related factors, such as age, education level, economic status, job status, marital status, and regular physical activity [33]. Marital status is a social support that allows people to feel cared for, supported, and affectionate through positive relationships with their families, including their spouses, and makes an individual feel worthwhile. The social support of a positive marital status has a positive effect on maintaining physical and emotional health and alleviates psychological distress [34]. In this study, marital status and the better the relationship was with their spouse and family, the higher their health-related quality of life. In the study by Kim [35], the depressive symptoms scores of divorced, separated, or widowed females were higher than that of females with a spouse, which is consistent with the results of this study. These results, along with the results of this study, indicate that the higher the depressive symptoms, the lower the health-related quality of life. A supportive marital status would reduce psychological distress and depressive symptoms and thus improve the health-related quality of life. Therefore, it is necessary to consider the support of the family to manage the health-related quality of life of middle-aged females.

The psychological distress, depressive symptoms, and emotional eating behaviors of middle-aged females affected their health-related quality of life. Depressive symptoms in middle-aged females are highly correlated with life stress, and the higher the life stress, the more severe the depressive symptoms [36]. Psychological distress and stress were found to be the strongest predictors of depressive symptoms in middle-aged females [37]. The rate of experiencing depressive symptoms among females aged 45–65 was found to account for 20.1% of all people experiencing depressive symptoms, indicating that depressive symptoms in middle-aged females are severe [38]. Uebelacker et al. reported that females who experienced many life-stress events had 4.02 times more depressive symptoms than females who did not, supporting the results of this study [34]. If depressive symptoms in middle-aged adults persist, it can negatively affect the health-related quality of life and life satisfaction by affecting various aspects, including emotional eating behaviors, fatigue, and physical problems, such as insomnia [39].

Traditionally, in a patriarchal society like Korea, the primary responsibilities of the family, such as the children’s education, family health, and relationships with the in-laws, are the female’s responsibility. These responsibilities are psychological distresses and can lead to depressive symptoms when the life stress is severe [40]. In the United States, middle-aged female depressive symptoms are 1.7 times higher than male depressive symptoms. This difference in depressive symptoms indicates that depressive symptoms among middle-aged females are a social problem [41]. These depressive symptoms in middle-aged females can lead to emotional eating behaviors [42].

Emotional eating behaviors refer to eating triggered by emotional cues and are generally defined as eating in response to negative emotions [43]. Christensen reported a cyclical relationship between an individual’s mental suffering and emotional eating behaviors [44]. The emotional eating behaviors of inappropriate eating due to a failure of self-control caused by negative emotions are a serious threat to a person’s health [16]. When a female has psychological distress and depressive symptoms related to negative life events, they exhibit emotional eating behaviors as an avoidant coping method [45]. Previous studies have reported that psychological distress, stress, and sadness were related to emotional eating behaviors [46, 47]. Therefore, identifying the causes of psychological distress and comforting and resolving the negative emotions are necessary [16].

This study verified the mediating effect of depressive symptoms and emotional eating behaviors on the relationship between psychological distress and health-related quality of life via a multiple mediation analysis. This study found that psychological distress had a direct effect on health-related quality of life. The increase in psychological distress was directly related to the deterioration of health-related quality of life. The reason for this is that negative emotions, such as depressive symptoms caused by psychological distress, are associated with emotional eating behaviors. This suggests that depressive symptoms and emotional eating behaviors due to psychological distress appear in a cyclical causal relationship, ultimately affecting health-related quality of life [48, 49]. Therefore, it is necessary to identify these factors related to psychological distress and health-related quality of life to understand the health-related quality of life of middle-aged females [50].

This study is meaningful since it was interested in the health-related quality of life of middle-aged females in Korea, the factors affecting their health-related quality of life were identified, and their mediating effect was confirmed. Therefore, if the psychological distress of middle-aged females is appropriately managed and their depressive symptoms are reduced with sufficient support from their family, their health-related quality of life can also be increased.

This study has a few limitations. First, it used a restricted sample resulting in limited generalizability. Second, this study examined the correlation between the variables; therefore, it is limited to identifying the cause and result of the fragmentary variables since an experimental test was not conducted to analyze the relationship between cause and effect. Third, since only the effects of depressive symptoms and emotional eating behaviors were analyzed to understand the relationship between psychological distress and health-related quality of life, the model could not identify the effects of various mediating variables. However, this study is significant because it provides evidence for improving health-related quality of life by identifying the relationship between psychological distress and health-related quality of life. Therefore, the health-related quality of life of middle-aged females can be improved by managing their psychological distress.

## Conclusion

In order to improve the health-related quality of life of middle-aged females, it is necessary to identify the factors that affect it. In this study, psychological distress, depressive symptoms, and emotional eating behaviors affected the health-related quality of life of middle-aged Korean females. It was also found that marital status affected the health-related quality of life. This study confirmed that psychological distress had a direct effect on health-related quality of life.

These findings serve as primary data for evidence-based intervention programs that alleviate emotional health problems, such as psychological distress and depressive symptoms in middle-aged females. Moreover, nurses can help develop effective treatment strategies to improve health-related quality of life by identifying and assessing potential symptoms of psychological distress, depressive symptoms, and emotional eating behaviors.

Additional studies are needed to identify the longitudinal influence of factors affecting the changes in the health-related quality of life of middle-aged females. In addition, studies are also needed to determine how the health-related changes in the quality of life in middle-aged adults affect their health and overall quality of life during old age.

## Data Availability

The datasets generated and/or analyzed during the current study are available from the corresponding author on reasonable request.
